# An Inverse Scaling Relationship between Stomatal Density and Mean Nearest Neighbor Distance: Evidence from a *Photinia* Hybrid and One of Its Parents

**DOI:** 10.3390/plants12213701

**Published:** 2023-10-27

**Authors:** Manli Sun, Ülo Niinemets, Qiying Li, Yabing Jiao, Weihao Yao, Peijian Shi

**Affiliations:** 1 Archives, Bamboo Research Institute, College of Ecology and Environment, Nanjing Forestry University, Nanjing 210037, China; sunmanli@njfu.edu.cn (M.S.); lqyin@njfu.edu.cn (Q.L.); ybjiao@njfu.edu.cn (Y.J.); whyao@njfu.edu.cn (W.Y.); 2 Institute of Agricultural and Environmental Sciences, Estonian University of Life Sciences, 51006 Tartu, Estonia; 3 Estonian Academy of Sciences, 10130 Tallinn, Estonia

**Keywords:** stomatal aggregation index, Chinese photinia, drought-tolerant plants, red tip photinia, regular distribution, spatial repulsion

## Abstract

Stomata are involved in transpiration and CO_2_ uptake by mediating gas exchange between internal plant tissues and the atmosphere. The capacity for gas exchange depends on stomatal density (SD), stomatal size, and pore dimensions. Most published work on stomatal quantification has assumed that stomatal distribution and stomatal density are spatially homogeneous across the leaf, but this assumption has been seldom tested. We selected 32 leaves from a *Photinia* hybrid, *Photinia × fraseri* ‘Red Robin’, and one of its parents, *P. serratifolia*. For each leaf, the leaf surface was divided into three or four equidistant layers along the apical–basal axis, and, in each layer, two positions, one closer to the midrib and the other closer to the leaf margin, were further selected. We calculated SD and mean nearest neighbor distance (MNND) for each lamina section and tested the scaling relationship between SD and MNND of the sampled stomatal centers using reduced major axis protocols. In addition, we calculated the stomatal aggregation index (SAI) for each lamina section to examine the spatial arrangement of stomata at the given size of field of view of 1.2 mm × 0.9 mm. We observed that SD decreased from the lamina apex towards the base for central lamina areas but varied little at leaf margins. An inverse scaling relationship between SD and MNND was observed for both species. This relationship could be used for SD estimation using the rapidly estimated trait, MNND. SAI did not vary significantly throughout leaf lamina, and the numerical values of SAI for all fields of view were greater than one, which indicates significant spatial repulsion between stomata. The study suggests that SD varies across leaf lamina to fine-tune plant water use and maximize carbon gain. However, spatial structures of stomata from different lamina sections exhibit similar patterns (i.e., spatial inhibition between stomata at small scales), probably due to hierarchical leaf vein patterns.

## 1. Introduction

Stomata function as gatekeepers, managing the fluxes of carbon dioxide and water vapor between the plant and the atmosphere and ultimately impacting the plants transpiration, respiration, and photosynthesis [1,2,3]. Stomatal conductance of fully developed leaves is very sensitive to rapid fluctuations in air humidity, light intensity, CO_2_ partial pressure, and air temperature [4,5,6,7]. Apart from instantaneous responses, maximum stomatal conductance depends on stomatal density and size (maximum pore dimensions), and leaves with a larger area of open stomatal pores have a higher stomatal conductance, leading to greater rates of photosynthetic carbon dioxide assimilation and causing transpiratory water loss [2,3,4,8,9,10]. Stomatal size and density are strongly affected by environmental conditions during leaf development, and such plastic responses ultimately determine maximum stomatal conductance [7,11]. Under unfavorable conditions during leaf growth and development, such as drought, leaves tend to have smaller stomata and greater number of stomata per leaf area [2,12]. This acclimation response allows the plant to fine-tune water use and increase water-use efficiency under drought.

Stomatal density (SD) is a trait that largely varies among species and has a strong environmental plasticity [4,10,11,13,14]. Current methods for calculating SD often hypothesize that there is no spatial variation in SD across the leaf. Therefore, measurements are usually limited to one sampling area per leaf [15,16]. However, some studies have shown that SD can vary considerably from one position to another on the same leaf [17,18]. In leaves of *Michelia cavaleriei* var. *platypetala* with hierarchical reticulate veins, stomata exhibit different spatial distribution characteristics at different spatial scales: regular distribution at small scales and random distribution at larger scales [17]. Shi et al. [18] showed that, for eight Magnoliaceae species, SD was characterized by high interspecific and intra-leaf variation. Given the spatial variation in SD and scale-dependent spatial aggregation, the size and position of the sampling area for SD estimation can lead to significant errors in stomatal conductance estimates based on SD and stomatal dimensions.

Several methods have been used to measure SD. Manual measurements using light microscopy are the traditional method of measuring SD [19,20]. However, this traditional method has many shortcomings, such as low computational efficiency—which limits the number of view areas analyzed—and susceptibility to human error. To address these limitations, some researchers have used a software, eCognition Developer 64, to carry out multi-scale segmentation of stomatal images to determine the best scale, shape, and compactness parameters to classify and extract stomata [21]. Although this method has a high accuracy, it is cumbersome to operate and places stringent requirements on the quality of microscopic images of leaf stomata. Based on the scaling relationship between SD and mean nearest neighbor distance (MNND), Shi et al. (2023) proposed a simple and convenient approach to estimate SD based on the scaling relationship between SD and MNND of sampled stomatal centers and successfully applied it to the calculation of the SD of eight Magnoliaceae species.

The *Photinia* genus belongs to the Rosaceae family, which has more than 60 evergreen and deciduous woody species, and the native *Photinia* species are distributed in eastern and southern Asia. Leaf margins for most *Photinia* plants have teeth and are seldom entire. Chinese photinia (*Photinia serratifolia* (Desfontaines) Kalkman), Japanese photinia (*Photinia glabra* (Thunb.) Maxim.), and their hybrid, red tip photinia (*Photinia × fraseri* ‘Red Robin’), are widely used as ornamentals in East Asia because of their drought tolerance. It is apparent that *P. × fraseri* is not a true “species” in the strict sense, but a hybrid. However, we have referred to it as a species for convenience hereinafter. The aim of this study is to determine whether SD varies horizontally (near the leaf margin versus near the midrib) and longitudinally within the leaf lamina of *P. × fraseri* and one of its parents, *P. serratifolia*. In addition, we asked whether the scaling relationship between SD and MNND for stomatal centers in a lamina section can be used as an alternative approach to estimate SD. The two species selected are widely applied in urban greening in China, because of their good characteristic of drought tolerance, and strongly contribute to the urban vegetation carbon sink for many Chinese cities [22,23].

## 2. Materials and Methods

### 2.1. Sampling Site

Mature undamaged leaves of two *Photinia* species, *P. × fraseri* ‘Red Robin’ and *P. serratifolia*, were collected from the campus of Nanjing Forestry University (118°48′35″ E, 32°04′67″ N; altitude of 23.7 m a.s.l.), Nanjing, Jiangsu Province, at 9:00 a.m. on 15 July 2022. The soil type in the sampling site is yellow brown soil. The mean annual precipitation of Nanjing is 1156 mm, and the mean annual temperature is 15.6 °C—according to the climate data collected between 1951 and 2014—which belongs to a typical subtropical monsoon climate [24]. The rainy season in Nanjing is from June to August, and the accumulated precipitation of the three months accounts for approximately 50% of the annual accumulated precipitation. For each species, 16 leaves were randomly selected from the intermediate positions of the canopies of ten *P. × fraseri* individuals and three *P. serratifolia* individuals.

### 2.2. Lamina Section Sampling

**Figure 1:**
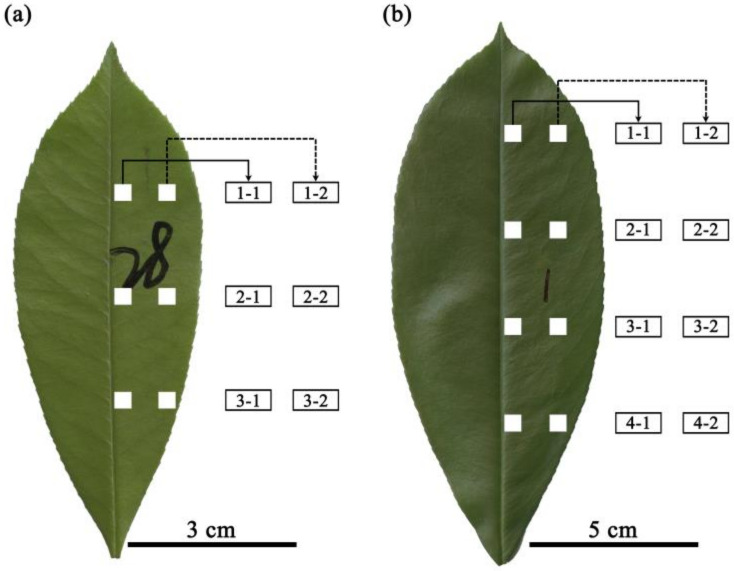
Positions of the selected leaf sections in *Photinia × fraseri* (**a**) and *P. serratifolia* (**b**). The lamina sections were equidistantly placed from lamina apex to base. In *P. × fraseri*, there were three layers from leaf apex to base and two positions from the midrib to the right leaf margin in each layer (six lamina sections per leaf). In *P. serratifolia*, there were four layers from leaf apex to base and two positions from the midrib to the right leaf margin in each layer (eight lamina sections per leaf). The numbers on the two leaves in panels (**a**,**b**) were marked to distinguish different leaves in the experiment for the following data analysis.

For each lamina, two longitudinal bands on the right side of the lamina were marked: one band closer to the midrib and the other closer to the leaf margin (Figure 1). Given the bilateral symmetry of the studied leaf, there is no need to sample lamina sections from the left side of the leaf. On each longitudinal band, three 0.3 cm × 0.3 cm lamina sections from the lamina apex to lamina base were selected in *P. × fraseri* and four 0.5 cm × 0.5 cm lamina sections in *P. serratifolia*. The lamina sections were located in equidistant intervals from lamina apex to base, and in each leaf, six sections were selected in *P. × fraseri* and eight sections in *P. serratifolia* (Figure 1). Larger lamina sections were used in *P. serratifolia* because this species has larger leaf blades, and the leaf veins were prone to damage in smaller sections.

Colorless nail polish was used to obtain stomatal imprints from each selected leaf section. Stomatal imprints were viewed with a Leica DM 2500 optical microscope (Leica Microsystems Shanghai, Shanghai, China) with a magnification of 10 × 10, and stomatal images were captured with the Leica microscope camera using LAS X software (version 3.4.2.18368; Leica Microsystems CMS GmbH, Germany) in the center of each lamina section (field of view of 1.2 mm × 0.9 mm) and saved as TIF files.

### 2.3. Extraction of Stomata and Planar Coordinates of Stomatal Centers

Photoshop software (version 13; Adobe Systems Incorporated, San Jose, CA, USA) was used to adjust the size and resolution of the stomatal image. A batch processing command was then used to convert all images from RGB to grayscale. We used the “readTIFF” function of the “tiff” package (version 0.1-11) [25] in R (version 4.2.0) [26] to read the stomatal images, manually select individual stomata using the “locator” function of the “graphics” package in R (version 4.2.0) [26], and extract the number and planar coordinates of the stomatal center in the observation window of [0, 1200 μm] × [0, 900 μm].

### 2.4. Calculation of Stomatal Density and Mean Nearest Neighbor Distance

Stomatal density (SD, namely, the number of stomata per unit lamina area) was estimated as the ratio of the number of stomatal centers to the area of the observation window (i.e., the field of view of 1.2 mm × 0.9 mm). For each observation window, 155 to 437 stomata were observed (see the online Supplementary Table S1). Using the “nndistG” function in the “splancs” package (version 2.01-42) [27] based on R (version 4.2.0) [26], we calculated the nearest neighbor distances for all points within any given observation window and then obtained the mean nearest neighbor distance (MNND). A total of 224 (96 for *P. × fraseri* and 128 for *P. serratifolia*) paired SD vs. MNND data were obtained.

### 2.5. Data Analysis

To test for the significance of the difference in SD and MNND between any two lamina sections of the same species, a linear mixed-effects model [28] was used. Layers or section positions were used as categorical fixed effects and leaves as random effects.

The composite index of Clark and Evans [29] is often used to study the spatial distribution patterns of stomata [30,31]. If stomata have a random spatial distribution, SD should scale inversely with the expected nearest neighbor distance (denoted as ${\bar{r}}_{E}$) [29]:

(1) $$\mathrm{SD}=\frac{1}{{4\bar{r}}_{E}^{2}}$$

Because SD was equal to the number of stomata per unit lamina area, ${\bar{r}}_{E}$ was then estimated to be the reciprocal of the square root of 4 × SD. The stomatal aggregation index (SAI) is defined as follows [29,31]:

(2) $$\mathrm{SAI}=\frac{\mathrm{MNND}}{{\bar{r}}_{E}}=2\times \mathrm{MNND}\times \sqrt{\mathrm{SD}}$$

In theory, the value of SAI ranges from 0 to 2.1491, with different values representing different spatial distributions of stomata [29]. The theoretical value of SAI = 0 implies that all individual stomata occupy the same position and maximum aggregation occurs; if 0 ≤ SAI < 1, the stomata are spatially aggregated; if SAI = 1, the stomata are randomly dispersed; and if SAI > 1, the stomata are regularly dispersed. We used reduced major axis protocols to estimate the intercept and slope of the linear regression equation for SD vs. MNND on a log–log plot [32,33]. The bootstrap percentile method [34,35] was used to calculate the 95% confidence intervals (CIs) of the intercept and slope for each species. For the two species (denoted as S_1_ and S_2_), we calculated 3000 estimates of the intercept and slope using the bootstrap method [34,35]. Denoting *D* as the difference in the replicates of the intercept between S_1_ and S_2_, we observed whether the 95% CI of *D* included 0. If the lower bound of the 95% CI of *D* is larger than 0, it indicates that the estimated intercept of S_1_ is larger than that of S_2_; if the upper bound of the 95% CI of *D* is smaller than 0, it indicates that the estimated intercept of S_1_ is smaller than that of S_2_; if the 95% CI of *D* includes 0, it indicates that there is no significant difference in the estimated intercepts between S_1_ and S_2_ (see ref. [35] for details). Theoretically, if the spatial dispersion of stomata is random, i.e., SAI = 1, then the 95% confidence interval for the intercept term of the regression line contains $\ln\frac{1}{4}$, while the 95% confidence interval of the slope of the regression line contains −2. It is worth noting that, in the actual experiment, the units of SD and MNND were mm^−2^ and μm, respectively, so the 95% confidence interval of the intercept here should contain $\ln\frac{1,000,000}{4}\approx 12.43$ [18]. All statistical analyses were carried out using R (version 4.2.0) [26].

The data of stomatal density, mean nearest neighbor distance, and aggregation index can be accessed in online Supplementary Table S1.

## 3. Results

**Figure 2:**
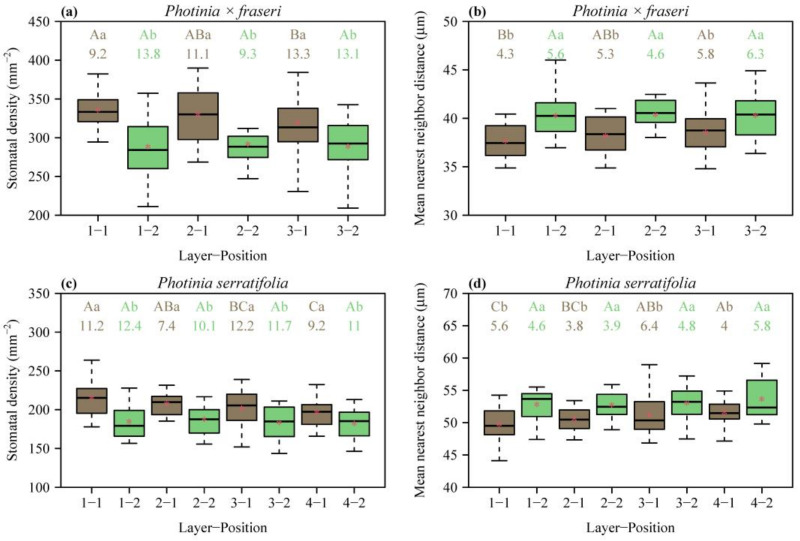
Box-and-whisker plots for leaf stomatal density (**a**,**c**), and mean nearest neighbor distance (**b**,**d**) for each position of each layer (Figure 1 for the definition of positions; *n* = 16 for each position). In the *x*-axis label, “Layer” represents the sampling direction from leaf apex to leaf base, and “Position” represents the sampling direction from the midrib to the right leaf margin. Uppercase letters at the top of the whiskers indicate the significance of differences between any two layers (from the leaf apex to leaf base) based on a linear mixed-effects model with the leaf number as a random effect. Lowercase letters show the significance of the difference between any two positions (the midrib vs. leaf margin, Figure 1) based on a linear mixed-effects model with the leaf number as a random effect. The numbers below the letters are the coefficients of variation (%) of stomatal SD or MNND. The segments in the boxes represent the medians, and the asterisks near the segments represent the means.

In each layer along the leaf (Figure 1), the SD was greater at the position closer to the midrib than at the position closer to the leaf margin (*p* < 0.05; Figure 2a,c). From the leaf apex to leaf base, SD values for the layers close to the leaf margin (i.e., position 2) did not differ significantly (Figure 2a,c). In contrast, for the layers close to the midrib of *P. × fraseri*, the SD of layer 1 was greater than that of layer 3 (*p* < 0.05, Figure 2a). For the layers close to the midrib of *P. serratifolia*, the SD of layer 1 was greater than that of layer 4 (*p* < 0.05, Figure 2c).

MNND was smaller at the position closer to the midrib than at the position closer to the leaf margin (*p* < 0.05; Figure 2b,d). From the leaf apex to leaf base, MNND values for the layers close to the leaf margin (i.e., position 2) did not differ significantly (Figure 2b,d). In contrast, for the layers close to the midrib of *P. × fraseri*, the MNND of layer 1 was smaller than that of layer 3 (*p* < 0.05, Figure 2b). For the layers close to the midrib of *P. serratifolia*, the MNND of layer 1 was smaller than that of layer 4 (*p* < 0.05, Figure 2d).

**Figure 3:**
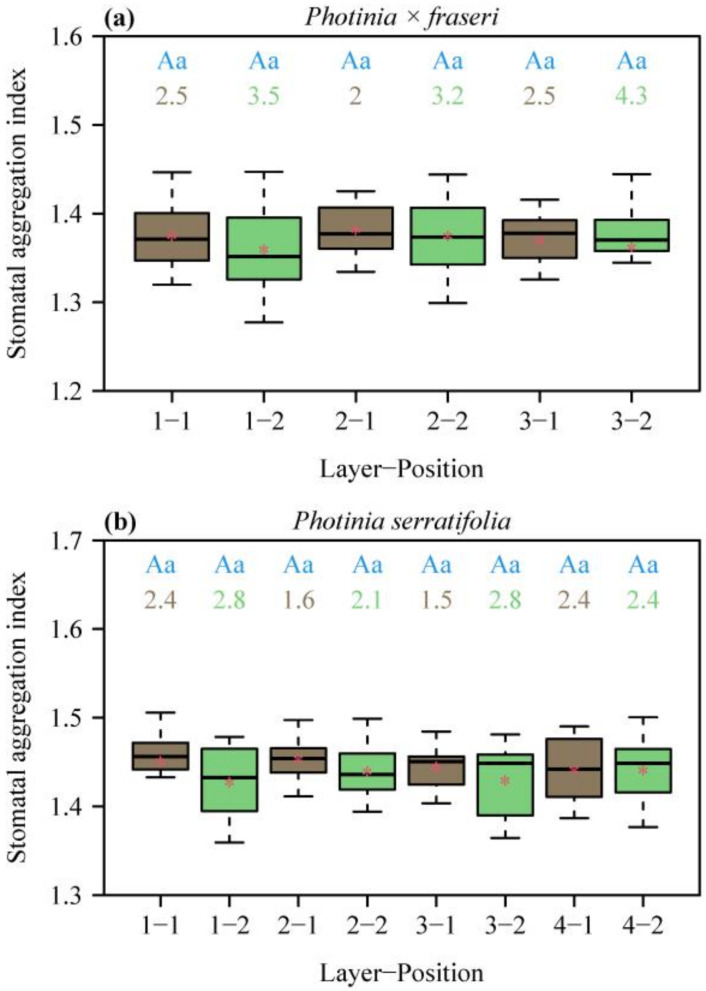
Box-and-whisker plots of stomatal aggregation indices (SAIs) for the two species, *P. × fraseri* (**a**) and *P. serratifolia* (**b**), for each position of each layer (Figure 1 for definition of positions, *n* = 16 for each position). In the *x*-axis label, “Layer” represents the sampling direction from leaf apex to leaf base, and “Position” represents the sampling direction from the midrib to the right leaf margin. Statistical significance is shown by uppercase and lowercase letters at the top of the whiskers, as in Figure 2. The numbers below the letters are the coefficient of variation (%) of SAI. The segments in the boxes represent the medians, and the asterisks near the segments represent the means.

All the aggregation indices (SAIs) for the two species were greater than unity, indicating a regular dispersion of stomata in each lamina section (Figure 3). SAI did not vary largely across different positions on the lamina (no significant effects of layers and positions on SAI). The numeric values of SAI > 1 suggest that there exists spatial repulsion (i.e., regular dispersion) at the given size of field of view of 1.2 mm × 0.9 mm.

**Figure 4:**
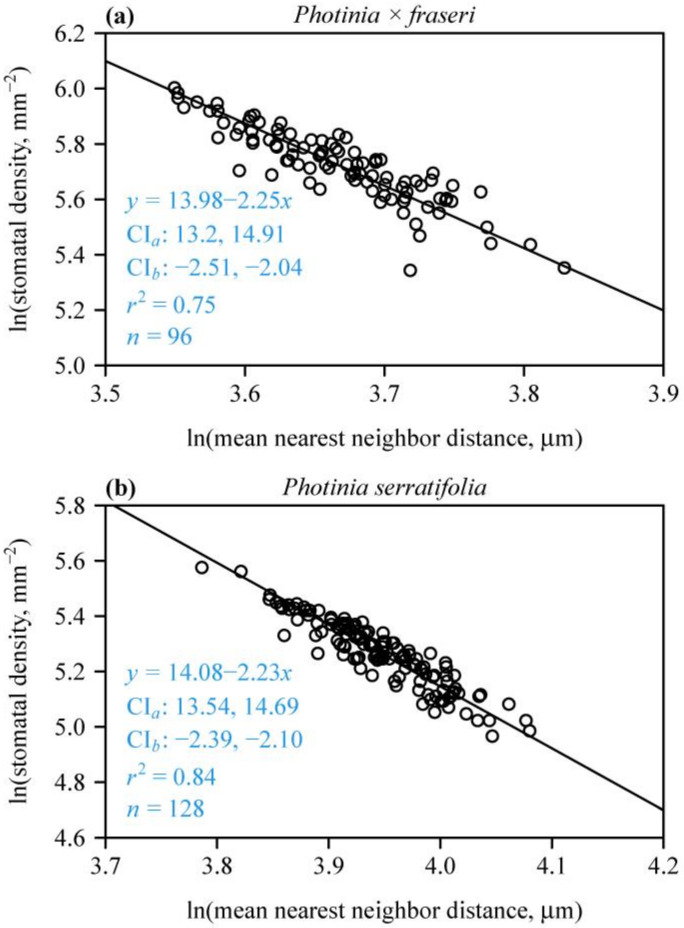
Fitted relationships of leaf stomatal density vs. mean nearest neighbor distance for *P. × fraseri* (**a**) and *P. serratifolia* (**b**) on a log–log scale. In each panel, the open circles represent the observations, and the straight line is the log–log regression curve; *n* is the sample size, i.e., the number of lamina sections; *r*^2^ is the coefficient of determination that reflects the goodness of fit of the linear regression; *x* is the natural logarithm of mean nearest neighbor distance of stomata in a field of view (in μm); *y* is the natural logarithm of the predicted stomatal density of a field of view (in mm^−2^); CI*_a_* provides the 95% confidence intervals of the intercept; and CI*_b_* provides the 95% confidence intervals of the slope.

For each species, there was a strong negative linear relationship between SD and MNND on a log–log scale (Figure 4), indicating that MNND could be used to estimate SD. In addition, neither of the two species had slopes with 95% CIs including –2. The corresponding 95% CIs of the intercepts did not include 12.43. These results are in agreement with SAI > 1 (Figure 3), further confirming that the stomata in the two *Photinia* species studied are not randomly dispersed. The 95% CI of the differences between the bootstrap replicates of the intercept of *P. × fraseri* and those of *P. serratifolia* were –1.08 and 0.918; the 95% CI of the differences between the bootstrap replicates of the slope of *P. × fraseri* and those of *P. serratifolia* were –0.294 and 0.244. Both 95% CIs included zero, which indicates that there was no significant difference between the estimated intercepts between the two species and the estimated slopes.

## 4. Discussion

Stomatal density (SD) exhibited a large spatial variation across a leaf, and the spatial arrangement of stomata is likely to be associated with the functions of leaves in photosynthesis and water evaporation. There was a negative scaling relationship between SD and the mean nearest neighbor distance (MMND) per field of view of 1.2 mm × 0.9 mm for each species, which tends to result from the spatial repulsion among stomata at small scales, and this can be potentially applied to the estimation of SD. We discussed the two topics in the following subsections.

### 4.1. Spatial Variation in Stomatal Density

Previous studies have overlooked spatial differences in stomatal density throughout the leaf, so we sampled multiple locations on one side of each of 32 leaves of two *Photinia* species to examine whether there were differences in the SD distribution. The data presented here indicate that SD exhibits a spatial variation across the leaf. Among the leaves sampled in this study, the mean SD of the lamina sections close to the leaf margin was lower than the mean SD of the lamina sections close to the midrib (Figure 2a,c). This is in agreement with our prior observations in eight Magnoliaceae species [17,18]. A probable explanation for this might be the interdependence of SD and vein density in regulating plant water status. Prior studies have demonstrated that SD and leaf vein density are positively correlated [10,36]. This coordination plays an important role in maintaining the dynamic balance between water loss and supply in plant leaves [10,36]. In addition, the average diameter of leaf veins near the midrib is larger than the average diameter of leaf veins near the leaf margin, implying that a greater water supply of leaf sections is matched by a greater SD [17,18,37,38].

We also observed that SD varied significantly from apex to base near the midrib, whereas the longitudinal variation was small near the leaf margin in two *Photinia* species (Figure 2a,c). From leaf apex to leaf base, there is a tendency for SD to decrease near the midrib. It seems to be possible that this result is due to the characteristics of the structure and function of the leaf lamina. Due to the unique morphological and structural characteristics of plant stomata, their development and physiological functions are very sensitive to environmental change [11,39]. The development of stomatal cells in different growth environments responds differently to environmental changes, such as drought, light, CO_2_, etc., to meet the growth and survival needs of the plant in different environmental conditions [40]. We argue that the decrease in stomatal density from the leaf apex to leaf base near the midrib reflects higher light availability at the lamina apex than at the lamina base. Higher light availability allows the plant to achieve, on average, a higher rate of photosynthesis, and, consequently, a greater SD near the leaf apex supports higher carbon dioxide diffusion into the leaf. The SD near the leaf margin, on the other hand, might not vary significantly from the leaf apex to leaf base because of higher level of stress encountered in the regions near the leaf margin, e.g., mechanical damage from the external environment, especially that caused by the feeding of herbivores [41,42]. In addition, due to low vein density and the dimensions at the leaf margins, apical increase in SD close to leaf edges might lead to excessive water loss. In conclusion, SD near the midrib of the leaf lamina varies greatly from leaf apex to leaf base, whereas SD near the leaf margin does not differ significantly from leaf apex to leaf base, reflecting a strategy to acclimate to the photosynthetic demands of different parts of the leaf and to reduce water loss by transpiration. This optimization of structure and function allows plants to photosynthesize efficiently and maintain water balance under different environmental conditions [43,44,45].

### 4.2. Spatial Repulsion between Stomata and Its Influence on the Estimation of Stomatal Density

Our data also revealed the presence of an inverse scaling relationship between SD and MNND in the two studied *Photinia* species. This finding aligns with the research conducted by [18], who investigated a negative scaling relationship between SD and MNND across eight Magnoliaceae species. The consistency of these findings in both the *Photinia* (Rosaceae) species and the Magnoliaceae species suggests that the inverse scaling relationship between SD and MNND may be a general pattern in plants with hierarchical reticular leaf veins. Understanding the mechanisms driving this relationship can shed light on the processes that shape stomatal distribution within leaves. Importantly, our study found no significant difference in the inverse scaling relationship between SD and MNND between the two *Photinia* species. The presence of strong correlations between SD and MNND suggests that in addition to direct measurements, an alternative approach can be used to estimate SD. This approach assumes that there is a spatial “repulsion” between stomata at small distances, resulting in a regular spatial distribution of stomata at the areole level; this pattern is likely to be valid for leaves from species with the same or similar venation patterns [17,18,46]. The observed inverse scaling relationship between SD and MNND suggests that stomata tend to exhibit a more regular arrangement at smaller scales, with a greater spatial inhibition between neighboring stomata. This suggests that stomata tend to position themselves further apart to uniformly support the leaf with carbon dioxide. This spatial organization may be influenced by factors such as cell signaling, developmental processes, or mechanical constraints. By utilizing this relationship, SD in species with similar venation patterns can be estimated without the need for labor-intensive stomatal counting. In summary, the confirmation of the inverse scaling relationship between SD and MNND in the two *Photinia* species strengthens the notion that this pattern may be a general phenomenon among plants. The concept of spatial repulsion between stomata provides a plausible explanation for this relationship. These findings have implications for estimating SD and understanding the ecological and evolutionary implications of stomatal distribution. Further research can delve into the functional and mechanistic aspects of this inverse scaling relationship across leaves of different venation and size.

## 5. Conclusions

There were significant differences in stomatal density (SD) and mean nearest neighbor distance (MNND) of stomata between different positions on the same leaf lamina in each of the two *Photinia* species. We found that SD close to the midrib was greater than that close to the leaf margin, and that SD increased from lamina base towards the apex in central lamina areas, but not at lamina margins. A strong inverse scaling relationship between SD and MNND was observed in both species. There was no significant difference in the slopes of SD vs. MNND on a log–log scale between the two *Photinia* species, and there was also no significant difference in the intercepts between the two *Photinia* species. However, the 95% confidence intervals of the two slopes did not include 2, and the 95% confidence intervals of the two intercepts did not include 12.43, which indicate that the spatial dispersion of stomata was not random in each of the two species but tends to exhibit spatial repulsion. In addition, the stomatal aggregation indices between different positions on the same leaf lamina in each of the two *Photinia* species did not differ significantly, and the stomatal aggregation index for each field of view of 1.2 mm × 0.9 mm was significantly greater than unity, which also constitutes evidence of the regular dispersion of stomata at the areole level. We argue that the spatial variation in SD and regular dispersion are important functional modifications allowing plants to optimize water use and photosynthesis across their leaf surface.

## Data Availability

The data can be found in the online Supplementary Table S1.

## Supplementary Materials

The following supporting information can be downloaded at: https://www.mdpi.com/article/10.3390/plants12213701/s1, Table S1: The raw data of stomatal density and mean nearest neighbor distance for the two *Photinia* species.
